# Accelerating medical education with ChatGPT: an implementation guide

**DOI:** 10.12688/mep.19732.2

**Published:** 2023-11-21

**Authors:** Justin Peacock, Andrea Austin, Marina Shapiro, Alexis Battista, Anita Samuel

**Affiliations:** 1 Department of Radiology and Radiological Sciences, Uniformed Services University, Bethesda, MD, USA; 2 Department of Military and Emergency Medicine, Uniformed Services University, Bethesda, MD, USA; 3 UHS Southern California Education Consortium, Temecula, CA, USA; 4 Center for Health Professions Education, Uniformed Services University, Bethesda, MD, USA

**Keywords:** ChatGPT, generative AI, artificial intelligence, medical education

## Abstract

Chatbots powered by artificial intelligence have revolutionized many industries and fields of study, including medical education. Medical educators are increasingly asked to perform more administrative, written, and assessment functions with less time and resources. Safe use of chatbots, like ChatGPT, can help medical educators efficiently perform these functions. In this article, we provide medical educators with tips for the implementation of ChatGPT in medical education. Through creativity and careful construction of prompts, medical educators can use these and other implementations of chatbots, like ChatGPT, in their practice.

## Introduction

November 30, 2022, has become a defining moment in the evolution of technology, when OpenAI ^©^ released ChatGPT to the public making artificial intelligence (AI) accessible to everyone. Within a week of its launch, ChatGPT had 1 million users, and 57 million users in the first month ^1^. The ease of use of ChatGPT and its broad information base has contributed to this exponential growth.

In recent years, chatbots powered by artificial intelligence (AI) have become increasingly popular in many industries, including healthcare. With the advent of advanced natural language processing techniques, chatbots converse with humans, providing personalized responses to their queries. In the field of medical education, chatbots can provide instant feedback, assistance, and information on a variety of medical topics. However, to make the most of these tools, it is essential to understand how to use them effectively. In this article, we provide tips on using ChatGPT, an advanced AI-powered chatbot, to enhance medical education. From using specific prompts to fine-tuning responses, we will explore the best practices for leveraging ChatGPT's capabilities in medical education.

### What is ChatGPT?

**Figure 1.**
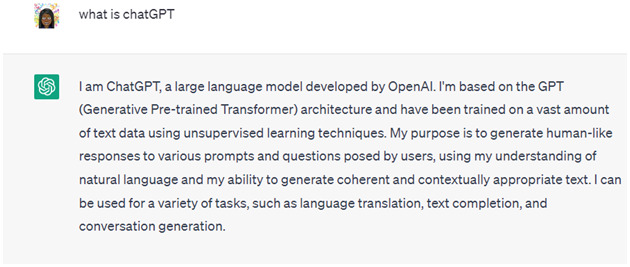
ChatGPT response to the prompt: “What is ChatGPT?”.

ChatGPT is a sophisticated chatbot that moves beyond the canned responses of traditional chatbots to provide more human-like responses to user queries. It uses a large language model (LLM), trained on vast amounts of data (around 3 billion words), with approximately 175 billion parameters ^1^. ChatGPT is built on the Generative Pre-trained Transformer (GPT) architecture and is categorized as “generative AI,” given its ability to generate new text in a conversational manner. The ChatGPT platform is continuously trained on the data users input into the system. ChatGPT has been used to write articles, summarize text, engage in conversation, translate text, generate code, and more. As an example, when asked “What is ChatGPT?”, Figure 1 provides the response that was generated by ChatGPT.

ChatGPT, released in November 2022, is based on GPT 3.5 technology. Since then, GPT 4 has been developed and released as ChatGPT-4 in April 2023. In this article, we limit our discussion to the freely available, older ChatGPT-3.5.

## ChatGPT in medical education

A study by Gilson *et al.* on the performance of ChatGPT in the USMLE Step 1 and 2 exams put ChatGPT front and center in medicine and medical education when ChatGPT performed above the 60% National Board of Medical Examiners (NBME) exam threshold ^2^. Since then, opinion pieces and commentaries have been written on how ChatGPT can be used in medical education ^3, 4^. Examples of ChatGPT’s use in medical education include producing simulation scripts, quizzes, personalized learning plans and much more, as detailed in this article.

The quality of ChatGPT output largely depends on the prompts (instructions) that the user inputs. Prompts are instructions provided to LLMs to “facilitate more structured and nuanced outputs ^5^.” Crafting an effective prompt for ChatGPT is a skill analogous to crafting a comprehensive search string for database searches. The sophistication of this skill has resulted in the emerging discipline of prompt engineering in which “carefully selected and composed sentences are used to achieve a certain” result ^6^. Within the current body of literature on using ChatGPT in medical education, there are few concrete examples of prompts or strategies for writing effective prompts to optimize ChatGPT output. In this article, we provide selected examples of how to use ChatGPT to enhance teaching, learning, and research in medical education.

Before we begin exploring ChatGPT implementation scenarios, it is important to know that ChatGPT is not perfect. It has, on average, an 85% accuracy rate in responses ^1^. ChatGPT was trained on specific types of data, with a greater focus on computer coding. Therefore, its accuracy across domains is variable and can result in ChatGPT generating false bibliographic citations, narrowly defined constructs, and incorrect mathematical calculations ^7, 8^. The concerning aspect of these errors is ChatGPT’s ability to be “confidently wrong” ^7^. Therefore, ChatGPT responses can act as a starting point, but they need to be rigorously checked and validated by the user. Additionally, it is important to re-inspect the prompts and information provided to ChatGPT to ensure that the data provided by the user is not erroneous or poorly constructed. This error can also result in ChatGPT generating incorrect answers.

### Implementation 1: creating a medical education curriculum

Curriculum development is a challenging process involving time, commitment, political capital, and patience, further exacerbated by a documented lack of training in curricula design ^9– 11^. Various materials need to be created, including needs assessment surveys, educational and assessment materials, and evaluation surveys, amongst others. ChatGPT can be used to assist in the development of a curriculum.

For example, we used a series of prompts to develop a Radiology residency diversity, equity, and inclusion (DEI) curriculum. We started by prompting ChatGPT to list potential curricular models for the proposed DEI curriculum. ChatGPT identified the Analyze, Design, Develop, Implement, and Evaluate (ADDIE), Kern, Accreditation Council for Graduate Medical Education (ACGME) Competency, and Culturally Responsive Curriculum models ^12– 15^. We then focused ChatGPT on the Kern model through prompting, and asked it to develop a comprehensive curriculum using the six steps in Kern’s model. Contextualizing to students and faculty provided more specific output. See Table 1 for an overview of prompts and ChatGPT responses.

**Table 1.** Prompts used to generate a curriculum.

Prompt provided	ChatGPT response
What models for medical education curriculum development would be most appropriate for a DEI curriculum in a Radiology Residency program?	Developing a curriculum on Diversity, Equity, and Inclusion (DEI) for a Radiology Residency program requires careful consideration of the appropriate models for medical education curriculum development. Here are some models that could be relevant:
I would like to develop a diversity, equity and inclusion curriculum for a group of Radiology residents using the Kern six step curriculum development model.	Great idea! Here's how you can use the Kern six-step curriculum development model to create a diversity, equity, and inclusion (DEI) curriculum for Radiology residents:
For Step 2 can you provide some survey questions that would allow for a targeted needs assessment of Radiology Residents about DEI topics?	Certainly! Here are some sample survey questions that could be used to conduct a targeted needs assessment of Radiology residents about DEI topics:
Can you provide examples of sample survey questions for the targeted needs assessment for Radiology faculty about DEI topics?	Certainly! Here are some sample survey questions that could be used to conduct a targeted needs assessment of Radiology faculty about DEI topics:
For Step 3, what would be some goals for the Radiology residency DEI curriculum?	Great question! Here are some goals that a Radiology residency DEI curriculum could aim to achieve:
What are examples of objectives for some of the goals that you identified for the DEI curriculum? Specifically, what objectives fit the SMART framework (i.e. specific, measurable, achievable, relevant, and time-bound)?	Certainly! Here are some examples of objectives that could align with the goals for a Radiology residency DEI curriculum that I outlined earlier, using the SMART framework:
For Step 4, what educational strategy would best address the goal of increase the cultural competence of Radiology residents to provide high-quality, patient-centered care to individuals from diverse backgrounds and the objective of within six months of completing the curriculum, Radiology residents will report an increased level of comfort in providing culturally sensitive care, as assessed by a post-curriculum survey.	To address the goal of increasing the cultural competence of Radiology residents and the objective of increasing their comfort in providing culturally sensitive care, a variety of educational strategies could be employed, such as:
Which of these educational strategies has been shown to be most effective in promoting cultural competence	Studies have shown that there is no single best educational strategy for promoting cultural competence, and the most effective approach will depend on the specific goals, needs, and learning styles of the participants. A combination of educational strategies is generally recommended to support the development of cultural competence.
For Step 5, what administrative items would we need to address to implement the DEI curriculum effectively in the Radiology residency program.	To implement a DEI curriculum effectively in the Radiology residency program, the following administrative items should be considered:
For Step 6, what questions could we ask to evaluate and gain feedback about the effectiveness of the DEI curriculum?	To evaluate and gain feedback about the effectiveness of the DEI curriculum in the Radiology residency program, the following questions could be asked:
DEI curriculum topics for a Radiology Residency program	Here are some specific DEI curriculum topics that a radiology residency program could consider incorporating into their training: These are just a few examples of DEI curriculum topics that could be incorporated into a radiology residency program. The specific topics and strategies used should be tailored to the unique needs and goals of the program and its residents.

See Supplement 1 in the extended data for the entire transcript.

While ChatGPT can provide a comprehensive curriculum, it cannot be used “as is.” User judgment is needed to identify relevant information and decide on what elements to explore further. Ethical curriculum developers should ensure that information obtained from ChatGPT is valid. However, careful use of the data obtained by ChatGPT can help to organize the development process and provide ideas and insights that might not have been thought about by the developer.

### Implementation 2: formulating and refining a course syllabus

After the development of the curriculum, ChatGPT can be used to formulate a new course syllabus or refine an existing syllabus. While the importance of effective course syllabi has been described in the literature, many are ineffective for learners, often omitting information and detail that will help learners succeed in the course, such as clear grading/assessment criteria, policies regarding course misconduct ( *i.e*., plagiarism), or the policy/procedure for syllabus changes ^16– 18^. For educators developing their first syllabus, ChatGPT can help them define essential components of a course syllabus by prompting: “ *What are the components of an effective medical school course syllabus?*” (see Supplement 2 in the extended data). ChatGPT can also be used to create a course syllabus by providing detailed prompts and descriptions of the planned course. For example, providing details such as the textbook, instructors, assignments, and medical school name generates a more relevant syllabus requiring less customization. The specific prompt we used was:

*Create a course syllabus for a pass/fail medical school course for 4th year medical students on geriatric medicine. The course consists of readings from "An Introduction to Geriatrics" by U.N. Panda. Additionally, there are four small group projects, and a final reflection paper. Additional activities include practicum learning at a nursing home, hospice facility, and Elderly community center. Please include standards of academic integrity, professionalism, and participation. The course is being designed for the Uniformed Services University medical school. Course instructors include John Smith, MD and Jane Doe, MD.*

Additionally, we used ChatGPT to generate a grading rubric for an assignment: “ *What would be the grading rubric for the final reflection paper?*” Some of the details need to be refined, and additional course details need to be provided, but ChatGPT can help generate the start of an effective course syllabus. With the right prompts, ChatGPT can help streamline course syllabus writing for the busy medical educator.

### Implementation 3: developing case scenarios and checklists for case-based or team-based learning

Case-based learning is an important educational strategy in medical education ^19– 22^. Case scenarios can be used in simulation, quality improvement, diversity and inclusion education, professionalism education, and educational research to prepare students for clinical practice ^22, 23^. A challenging task for medical educators is developing compelling, case-based or team-based learning activities. ChatGPT can help develop these scenarios or give ideas for scenarios that educators or researchers have not considered.

The quality of the case scenario developed depends on the specificity of the prompt provided to ChatGPT. For example, if one wanted to develop a simulation case scenario that assesses the AAMC Intrapersonal Competency: Ethical Responsibility to Self and Others in fourth-year medical students, a generic prompt to ChatGPT of “ *Create a case scenario*” results in a fictional scenario about “Jane’s Job Interview” (see Supplement 3 in the extended data). Further prompting to “ *Help me develop a medical professionalism case scenario.*” results in a “difficult patient” scenario with a patient seeking more pain medication. Asking ChatGPT to “ *develop a simulation-based case scenario for medical students on AAMC intrapersonal competencies*” revealed that ChatGPT was unfamiliar with the AAMC competencies. Further refining the prompt by asking ChatGPT to develop a scenario that assesses specific AAMC competencies and provides the description of the competency from the AAMC website results in a well-crafted scenario assessing a student’s ability to identify a mistake and appropriately acknowledge and remedy the error in a professional manner. Through careful prompting, ChatGPT can help educators and researchers develop effective case scenarios for a variety of educational experiences ^24, 25^.

ChatGPT can also help create checklists for simulation scenarios that can be used to assess learners and provide an effective debrief ^25^. In a dynamic simulation, it is important for evaluators to have efficient and standardized checklists or assessment tools to help them focus their attention while assessing participants ^26– 28^. Furthermore, ChatGPT maintains a history of chat interactions, making it possible to build on previous prompts eliminating the need to repeat contexts. The prompt “ *What would be an appropriate simulation checklist for this scenario to assess competence?*” automatically references the previous context and responds “ *Here is a possible simulation checklist that could be used to assess the competence of fourth-year medical students in demonstrating the AAMC ethical responsibility to self and others core competency in the scenario:*” ChatGPT appropriately identified components of the simulation that would help identify competence, including acknowledgement of the mistake, identification of the error, reflection on the mistake, encouraging others to be honest, and cultivating personal and academic integrity. ChatGPT further identified specific actions that the participant might take for each of these key principles.

### Implementation 4: designing knowledge check assessments

ChatGPT can be used to develop quiz questions to facilitate assessment ^25^. Appropriate quiz questions can be generated by training ChatGPT on sample questions for the desired exam and by providing feedback to responses that ChatGPT provides in response to the questions. For example, we provided ChatGPT with example NCLEX questions ^29^. ChatGPT got 50% of the questions correct (see Supplement 4 in the extended data). ChatGPT was able to correct its response to one question on the second try. For a question on the purpose of defibrillation, ChatGPT responded with an answer that was outside the response choices provided. It could not understand the correct answer of “cause asystole so the normal pacemaker can recapture” and kept repeating that defibrillation’s purpose was to restore a normal cardiac rhythm. It is critical to remember that ChatGPT does make errors and provides erroneous information ^3, 30– 32^. So, verifying the material provided by ChatGPT is vital.

After training ChatGPT on sample questions, we prompted it with: “ *After reviewing the provided example NCLEX exam questions, please design a similar sample multiple choice question in the style of the NCLEX exam with answer explanation*.” ChatGPT developed an assessment along with feedback on the correct response. Asking for additional questions tended to cause ChatGPT to ask similar questions ( *i.e.*, medication-based questions). Prompts needed to be refined to other contexts such as non-medication questions and specifically posed as “ *Please provide a non-medication based NCLEX question*.” While medical educators can use this process to generate knowledge check questions, medical students can also use this process to generate practice questions. Quiz sets such as this can be helpful practice sets for medical school students.

### Implementation 5: applying education theory to practice

Accreditation Council for Graduate Medical Education (ACGME) training standards and evidence-based medicine have increased interest in medical education practice informed by educational theories ^33^. Principles of educational theory can help provide structure and theoretical support for innovations in medical education. ChatGPT can help to describe educational theories and apply the principles of those theories to practical medical education.

We utilized ChatGPT to define Activity Theory and describe the principles of Activity Theory (see Supplement 5 in the extended data). It provided a summary of essential points of the theory and some common principles of the theory. We then asked ChatGPT, “ *How can I apply activity theory in a medical student course about anatomy?*” It offered suggestions such as including goal-directed learning activities ( *a lab activity where students have to identify and label different anatomical structures*), mediating tools and symbols ( *anatomical models or 3D visualizations to help students visualize and understand the location and function of different structures*), encouraging social learning, highlighting the history and cultural aspects of Anatomy, and emphasizing the interconnected, systemic nature of the different organ systems and body parts. The broad nature of these responses reflects the broad prompt provided to ChatGPT. As in Implementation 1, more detailed and narrowed prompting of ChatGPT would have led to a more focused response tailored to the specific question of interest. Despite its limitations, ChatGPT can provide a practical starting point for medical educators to get started with teaching based on educational theories.

### Implementation 6: crafting personalized learning plans to address deficiencies

ChatGPT can be utilized to develop questions about a topic or build questions based on specific course objectives. Using these questions, a learner can ask ChatGPT to help craft learning plans, topics, and resources. Individualized learning plans are an effective developmental and assessment tool for achieving higher levels of medical proficiency ^34– 38^. ChatGPT can help learners and educators enhance self-regulation and deliberative practice in a structured, goal-oriented manner.

Utilizing the learning objectives from a publicly available medical microbiology course description from the University of Cincinnati ^39^, we asked ChatGPT to design questions that would test knowledge about those course objectives, *“I am taking a medical school course in microbiology, I would like to develop questions to test my knowledge of the course learning objectives and develop a personalized learning plan to address deficiencies in my knowledge.”*(See Supplement 6 in the extended data) After refining the questions, we assessed a hypothetical deficiency in antifungal medications. We asked ChatGPT to design a learning plan, topics, and resources to correct the deficiency. ChatGPT referred the learner to helpful resources to address the identified deficiency. Two points to keep in mind are to: (1) check the references provided and (2) the more specific and detailed the prompt, the better the response.

### Implementation 7: evaluating and revising written work, including reports, essays, manuscripts, or written responses

One of the controversies surrounding ChatGPT is that of academic integrity. Educators and researchers fear that ChatGPT could be used to generate written work for courses or publications. Publishers are working on policies regarding the usage of ChatGPT in publications ^40, 41^. While this is a genuine concern, ChatGPT is a powerful tool for evaluating and providing critical feedback on the organization and quality of written work.

We used ChatGPT to review a manuscript, evaluate quality, and provide suggestions for improvement ^42^. The conversation with ChatGPT was initiated with the prompt, “ *I am writing a manuscript and would appreciate your feedback on components of the manuscript*.” A challenge with providing the manuscript to ChatGPT is the size limitations for the prompts (approximately 500 words), so the manuscript was input into ChatGPT in sections ( *e.g*., abstract, introduction). The ChatGPT responses accurately identified areas for improvement and provided suggestions for improvement (see Supplement 7 in the extended data). Since the manuscript was provided in sections, a few ChatGPT responses were discrepant or addressed in later sections of the manuscript. To address this, after entering the segmented manuscript into ChatGPT, we asked it to evaluate and provide suggestions for the whole manuscript, which resulted in a succinct list of suggested improvements.

Used ethically, ChatGPT can function as an effective reviewer with a helpful set of AI eyes. It is important to note that any information input in ChatGPT becomes part of the ongoing training for the program. Consequently, one must be very careful not to submit another’s work without express permission, as it could be breaching ethical principles or copyrights ^32, 43, 44^. There is also the potential for ChatGPT to help authors with different language backgrounds correct grammatical and other language errors ^45^.

### Implementation 8: summarizing complex articles or data sources that are readily available

An important part of clinical practice is staying abreast of new guidelines, governmental mandates, and discoveries. In addition to being time-consuming, documents such as government mandates can be challenging to understand. ChatGPT can help by quickly and efficiently providing summaries of websites or documents, which can be help gain insight into complex topics such as health care bills ^32^.

We asked ChatGPT to look at the Consolidated Appropriations Act 2021 (House Bill 133) and summarize the information in that bill. We further asked it to summarize information about healthcare funding in the bill and the No Surprise Act (see Supplement 8 in the extended data). ChatGPT was able to provide useful summaries, but required prompting to dig deeper into the material. Whether it is an article, a government bill, a theoretical concept, or another piece of information, if it is accessible by the internet, then ChatGPT can analyze, summarize, and thematically categorize data.

### Implementation 9: enhancing research

ChatGPT is effective in enhancing various aspects of qualitative research. We input interview transcripts into ChatGPT and prompted ChatGPT with “ *What are the themes in this text?*” In less than 10 seconds, ChatGPT generated a series of topics and subtopics. ChatGPT could also provide a summary of the transcript. As with all ChatGPT responses there was a margin of error. While ChatGPT is not to be used in lieu of human coding, it is helpful when used to verify human coding.

Another aspect of research is making connections between concepts. We used ChatGPT to tease apart nuanced differences between several constructs ( *i.e*., facilitation, scaffolding, cues, cueing) to guide the development of decision support guidelines for full-text article screening for a systematic review. During title and abstract screening in a literature review, we noticed that the above constructs were the most commonly mentioned; however, they were rarely defined and infrequently included deep descriptions. Therefore, we started with the construct of 'facilitation in education' and then prompted ChatGPT to elaborate on facilitation specifically related to simulation-based learning to identify similarities and differences and prompted ChatGPT with " *How is facilitation defined in healthcare simulation?"* We continued by asking ChatGPT to compare the other related terminologies ( *e.g*., scaffolding, cues, cueing) with each other and provide references. We reviewed the responses, references, and our theoretical framework of scaffolding to construct a series of if/then statements that we could use to guide full text screening of articles in a literature review (see Supplement 9 in the extended data).

### Implementation 10: developing proposals for medical institutions, medical societies, or other organizations

ChatGPT can be used to generate various documents that a medical educator or researcher might need to create during their career. For example, we used ChatGPT (see Supplement 10 in the extended data) to create a proposal for change in the organizational structure of a professional medical society. The prompt detailed a plan to dissolve overlapping committees with low participation and open up the activities of those committees as micro-volunteering opportunities for the whole society. The prompt also proposed a points system to motivate participation in the micro-volunteering opportunities. ChatGPT developed a well-organized proposal about the concerns, the proposed changes, and the potential benefits.

ChatGPT can help to eliminate some of the entry-level barriers to many documents that are daily components of medical education and research. Ethical use of ChatGPT requires that users provide appropriate prompts, evaluate the output for accuracy, and do not just copy and paste from the ChatGPT output. Rather, users need to use ChatGPT output as a starting point and edit and personalize as necessary.

### Implementation 11: developing empathetic and comprehensive solutions to programmatic challenges

Medical education programs and departments experience varied problems and challenges that necessitate empathetic and complete responses and solutions. These problems can range from developing comprehensive longitudinal educational programs to classroom management, human resource problems, and challenging patient interactions. Recent research has demonstrated that ChatGPT gives higher quality and more empathetic responses to patient questions than physicians ^46^. The authors hypothesized that with the rise of virtual healthcare, ChatGPT could improve patient satisfaction, manage increased workloads, and reduce provider burnout. We used ChatGPT to perform a range of functions in a simulation program, including developing an initial framework for a simulation budget, generating a prioritized list of simulators available for fundamental laparoscopic surgery skills based on fidelity and cost, and even a list of ideas for how to respond appropriately to learners who will not put away their phones during lecture (see Table 2). The initial responses provided an excellent first draft to address a nuanced issue. Although ChatGPT can provide empathetic responses, it could also provide responses that are considered unprofessional and biased and need careful consideration before implementation ^32^.

**Table 2.** Prompts used to develop solutions to programmatic challenges.

Prompt provided	ChatGPT response sample
What are several ways to kindly ask someone to put away their phone during a lecture?	When requesting someone to put away their phone during a lecture, it's important to approach the situation politely and respectfully. Here are several ways you can kindly ask someone to do so:
How do you create a budget for a simulation center?	Creating a budget for a simulation center involves careful planning and consideration of various factors. Here's a step-by-step guide to help you through the process:
Explain how a simulation center can ensure appropriate diversity in simulators	To ensure appropriate diversity in simulators, a simulation center can take several steps:

See Supplement 11 in the extended data for complete script

### Implementation 12: developing reference lists for grants, manuscripts, or other written documents

Literature searches are a crucial part of research. It does, however, take time. We used ChatGPT to generate references for various topics including “ *Create a reference list for articles about the SARS-CoV-2 lung infection that highlights important radiologic imaging findings*” and “ *Create a reference list for articles about DEI in Radiology.*” ChatGPT is set up to generate ten results by default. We found that ChatGPT is good at creating references or resources for certain topics and not for others. In response to the SARS-CoV-2 lung infection prompt, ChatGPT generated ten high-impact articles from the Radiology literature about the imaging findings from the virus. Conversely, for the prompt on DEI in Radiology, ChatGPT generated ten article references, nine of which were fictitious, and one was unrelated to the requested topic. Researchers have found that the amount of errors in ChatGPT responses is indirectly correlated with the volume of literature available on the topic, which is likely the reason for the above finding by the authors ^47^. As with all ChatGPT responses, the references generated by ChatGPT need to be checked to determine their authenticity. In addition to providing incorrect references, ChatGPT 3.5 does not include recent references. ChatGPT 3.5’s data set ends in 2021; hence newer data is not included.

## Conclusion

The possibilities for ChatGPT use in medical education are endless and limited only by the user’s imagination. ChatGPT can serve medical educators, learners, and researchers in their various tasks. ChatGPT can generate text, translate languages, write different kinds of creative content, and answer questions in an informative way. However, it is important to remember that ChatGPT is not a human and does not have the same level of understanding as a human. It is also essential to use ChatGPT in a way that is consistent with personal teaching styles and goals. ChatGPT can be a helpful tool for providing students with personalized feedback and support. However, it is crucial to use ChatGPT in a way that does not replace human interaction. Lastly, it is important to realize that many of the proposed benefits of ChatGPT in medical education are unstudied, and without data it is difficult to assess their validity or benefit.

With these advances, developing an ethical and methodical approach is critical ^4, 32^. Ethical concerns include authorship, plagiarism, copyright infringement, false information utilization, and inherent bias built into the model ^4, 32, 43, 44^. For example, ChatGPT should not be used to provide medical advice or diagnosis or to create harmful or offensive content. Overall, ChatGPT can be a helpful tool for medical education. However, it is important to use ChatGPT in a safe, ethical, and consistent way. Some ways to accomplish this include rigorous review of the outputs from ChatGPT prior to publication or wider distribution, inputting one’s own work and not that of others, checking for inherent biases in the outputs, and integrity regarding the source of produced work ^32, 43, 44^.

In addition to carefully crafting prompts with sufficient contextual details to ensure high-quality outputs, verifying all output information that ChatGPT generates is key to effectively using ChatGPT.

As we use ChatGPT, we must be cognizant of the limitations of the technology ^3, 30, 32^.

- ChatGPT has only been trained on data till 2021 ^48^.
- ChatGPT has been trained on human-created data. Therefore, the biases and inaccuracies within the data are repeated by ChatGPT.
- ChatGPT has been trained extensively on certain domains, such as computer languages. It was not trained equally on data such as crochet patterns. Therefore, responses generated by ChatGPT in these domains have a larger inaccuracy rate ^49^.

ChatGPT and similar AI-based technologies like Bard (Google) and Bing (Microsoft) are the future. These technologies can potentially accelerate innovation in medical education and clinical practice. Learning to harness these technologies and using them effectively can enhance the efficiency of medical educators, learners, and researchers.

## Disclaimers

The opinions and assertions expressed herein are those of the author(s) and do not necessarily reflect the official policy or position of the Uniformed Services University or the Department of Defense or the Henry M. Jackson Foundation.

## Data Availability

Zenodo:. Accelerating medical education with ChatGPT: An implementation guide. https://doi.org/10.5281/zenodo.8178876 ^50^ The project contains the following underlying data: 12 Supplement files labeled [Supplement 1.docx] through [Supplement 12.docx] (ChatGPT prompts and discussions for the 12 implementations discussed in the article). Data are available under the terms of the Creative Commons Attribution 4.0 International license (CC-BY 4.0).
