# Supplementary material for: Accelerating medical education with ChatGPT: an implementation guide
Source: MedEdPublish (2016). 2023 Nov 21;13:64. Originally published 2023 Sep 1. [Version 2] doi: 10.12688/mep.19732.2 (PMC10910173; doi:10.12688/mep.19732.2)
Supplement: Peer review history

# Reviewer report: Reviewer response for version 2

**DOI:** 10.21956/mep.21461.r35413

**Published:** 2024-03-03

**Authors:** Surapaneni Krishna Mohan

**Affiliations:** 1 Panimalar Medical College Hospital & Research Institute, Poonamallee, India

The article thoroughly explores various applications of ChatGPT in medical education, providing concrete examples and implementation strategies. This depth enhances its practical relevance for educators. The article also effectively highlights the potential benefits of using ChatGPT in curriculum development, syllabus creation, case scenario development, and other aspects of medical education. This relevancy strengthens its appeal to educators. Furthermore, the article maintains a clear and organized structure, presenting a range of implementations with specific prompts. This clarity aids educators in understanding how to practically integrate ChatGPT into their teaching methods.

**Revisions:**

1. The inclusion of visual aids, such as flowcharts or diagrams illustrating the implementation process, could enhance the article's visual appeal and make complex concepts more digestible. 
2. While the article rightly acknowledges the impact of ChatGPT in medical education, it would benefit from suggesting areas for future research or proposing methodologies for assessing the technology's efficacy in educational settings.

If evidence from practice is presented, are all the underlying source data available to ensure full reproducibility?

Yes

Is the topic of the practical tips discussed accurately in the context of the current literature

Yes

Are all factual statements correct and adequately supported by citations?

Yes

Are the conclusions drawn balanced and justified on the basis of the presented arguments?

Yes

Are arguments sufficiently supported by evidence from the published literature and/or the authors’ practice?

Yes

Reviewer Expertise:

Medical education, assessment, teaching-learning methodologies, artificial intelligence, gamification,

I confirm that I have read this submission and believe that I have an appropriate level of expertise to confirm that it is of an acceptable scientific standard, however I have significant reservations, as outlined above.

# Reviewer report: Reviewer response for version 2

**DOI:** 10.21956/mep.21461.r35319

**Published:** 2023-11-28

**Authors:** Xi Lin

**Affiliations:** 1 East Carolina University, North Carolina, USA

The authors well addressed the suggestions.

If evidence from practice is presented, are all the underlying source data available to ensure full reproducibility?

Not applicable

Is the topic of the practical tips discussed accurately in the context of the current literature

Yes

Are all factual statements correct and adequately supported by citations?

Yes

Are the conclusions drawn balanced and justified on the basis of the presented arguments?

Partly

Are arguments sufficiently supported by evidence from the published literature and/or the authors’ practice?

Yes

Reviewer Expertise:

Distance education, online learning, game-based learning, Artificial Intelligence.

I confirm that I have read this submission and believe that I have an appropriate level of expertise to confirm that it is of an acceptable scientific standard.

# Reviewer report: Reviewer response for version 1

**DOI:** 10.21956/mep.21140.r34888

**Published:** 2023-10-18

**Authors:** Ken Masters

**Affiliations:** 1 Medical Informatics, Medical Education Unit, Sultan Qaboos University, Muscat, Oman

Overall, a useful paper.  It is a pity that the authors have limited the work to ChatGPT 3.5, but I can understand that many teachers will not have access to the paid version, so would first wish to use 3.5 only.

 Some issues, which are relatively easily addressable, are:

- “It is a large language model” should rather be “It uses a large language model”
- One thing you may wish to consider: when ChatGPT answers questions incorrectly, it is a good idea to re-inspect the question and the options.  It is possible that the fault does not lie with ChatGPT, but rather, with the material.
- One of the things you demonstrated in Implementation 1 is that the user should refine and re-prompt in order to get to details, because, frequently, a very broad question will result in very broad answers only.  But this should be raised in Implementation 5, where the question “How can I apply activity theory in a medical student course about anatomy” is extremely broad, and so has a very broad answer.  In order to implement anything of value, the user should drill down and interrogate ChatGPT with further prompting.
- On Implementation 7: although you mention that it should be used ethically, you need to consider the issues here a little more deeply.  By default, OpenAI grants itself the right to use any submitted materials as part of its training.  If you are submitting your own work, that is fine, but, if you are submitting someone else’s work (including student work), you may be breaching ethical principles.  It might be worthwhile to refer to some of the literature on the ethics of AI in medical education.

On the whole, a good read, and I look forward to seeing the revised version.

If evidence from practice is presented, are all the underlying source data available to ensure full reproducibility?

Yes

Is the topic of the practical tips discussed accurately in the context of the current literature

Yes

Are all factual statements correct and adequately supported by citations?

Yes

Are the conclusions drawn balanced and justified on the basis of the presented arguments?

Yes

Are arguments sufficiently supported by evidence from the published literature and/or the authors’ practice?

Yes

Reviewer Expertise:

Medical Education; Medical Informatics

I confirm that I have read this submission and believe that I have an appropriate level of expertise to confirm that it is of an acceptable scientific standard, however I have significant reservations, as outlined above.

# Response

**Published:** 2023-11-16

**Authors:** Justin Peacock

**Affiliations:** Department of Radiology, Uniformed Services University, Bethesda, MD, USA

November 6, 2023   Editorial Staff *MedEdPublish*   Dear Editorial Staff,   Please find our revised manuscript “Accelerating medical education with ChatGPT: An implementation guide” enclosed for publication in the *MedEdPublish*.  We are grateful for the review of our work and assistance in improving its quality.   The article has been revised as detailed below.  

*Point 1. Limitation in the use of ChatGPT 3.5.* We agree with the reviewer that ChatGPT 4 is likely to provide a more high-quality product and would result in different outcomes. However, as noted, it costs, and therefore limits the use among educators.  

*Point 2. “It is a large language model” should rather be “It uses a large language model”*   We agree with the reviewer and have made the change in the text.  

*Point 3. When ChatGPT answers questions incorrectly, it is helpful to assess the prompt and see if the fault lies in the prompt.*   We agree with the reviewer that this is a possibility and have added a statement addressing this at the end of the Introduction:   “Additionally, it is important to re-inspect the prompts and information provided to ChatGPT to ensure that the data provided by the user is not erroneous or poorly constructed. This error can also result in ChatGPT generating incorrect answers.”**

*Point 4. Address refinement and re-prompting considerations in Implementation 5, which contains a broad prompt.*   We agree that the Implementation 5 prompt is broad and have added the following text to that section:   “The broad nature of these responses reflects the broad prompt provided to ChatGPT. As in Implementation 1, more detailed and narrowed prompting of ChatGPT would have led to a more focused response tailored to the specific question of interest.”    

*Point 5. Address the ethical concerns more deeply in Implementation 7, including literature.*   We agree that this needs to be further addressed in Implementation 7, we have added the following to that section:   “It is important to note that any information input in ChatGPT becomes part of the ongoing training for the program. Consequently, one must be very careful not to submit another’s work without express permission, as it could be breaching ethical principles or copyrights ^49-51^.”

# Reviewer report: Reviewer response for version 1

**DOI:** 10.21956/mep.21140.r34890

**Published:** 2023-10-18

**Authors:** Xi Lin

**Affiliations:** 1 East Carolina University, North Carolina, USA

The topic related to ChatGPT is quite popular, and this article contributes to the use of ChatGPT in medical education. Overall, the implementations of ChatGPT are well-written with detailed information. I have two suggestions:

1. It appears that the authors discussed how they utilized ChatGPT for various activities. However, I wonder if the outcomes of ChatGPT services indeed benefit educators, learners, and researchers. While ChatGPT can serve them in various tasks, it would be beneficial to hypothesize that ChatGPT may be a helpful tool, but without data, we cannot be certain of its impact. Therefore, the authors may consider discussing this in the conclusion section. 
2. I recommend expanding the conclusion section to address more limitations or challenges associated with using ChatGPT in medical education. For instance, mentioning the importance of using ChatGPT in a safe, ethical, and consistent manner is a good point. The authors may provide more insights on how to achieve this.

Overall, it is a great study.

If evidence from practice is presented, are all the underlying source data available to ensure full reproducibility?

Not applicable

Is the topic of the practical tips discussed accurately in the context of the current literature

Yes

Are all factual statements correct and adequately supported by citations?

Yes

Are the conclusions drawn balanced and justified on the basis of the presented arguments?

Partly

Are arguments sufficiently supported by evidence from the published literature and/or the authors’ practice?

Yes

Reviewer Expertise:

Distance education, online learning, game-based learning, Artificial Intelligence.

I confirm that I have read this submission and believe that I have an appropriate level of expertise to confirm that it is of an acceptable scientific standard, however I have significant reservations, as outlined above.

# Response

**Published:** 2023-11-16

**Authors:** Justin Peacock

**Affiliations:** Department of Radiology, Uniformed Services University, Bethesda, MD, USA

Please find our revised manuscript “Accelerating medical education with ChatGPT: An implementation guide” enclosed for publication in the *MedEdPublish*.  We are grateful for the review of our work and assistance in improving its quality.   The article has been revised as detailed below.  

*Point 1. Relay uncertainty in usefulness of ChatGPT in medical education in the Conclusion.*   We agree that many of the proposed benefits and applications of ChatGPT in Medical Education are unstudied. We have reflected this uncertainty of outcome in the Conclusion as follows:   “Lastly, it is important to realize that many of the proposed benefits of ChatGPT in medical education are unstudied, and without data it is difficult to assess their validity or benefit.”    

*Point 2. Expand the Conclusion to address more limitations or challenges associated with ChatGPT in medical education.* We agree that a broader description of the limitations or challenges to the use of ChatGPT in medical education is warranted and have addressed this in the Conclusion as follows:   We have added sentences to paragraph 2 of the Conclusion that address the ethical challenges of using ChatGPTand ways of improving ethical use of ChatGPT.
